# Strategies to Counteract Oxidative Stress and Inflammation in Chronic-Degenerative Diseases

**DOI:** 10.3390/ijms23126439

**Published:** 2022-06-09

**Authors:** Cecilia Prata, Tullia Maraldi, Cristina Angeloni

**Affiliations:** 1 Department of Pharmacy and Biotechnology, Alma Mater Studiorum—University of Bologna, Via Irnerio 48, 40126 Bologna, Italy; 2 Department of Biomedical, Metabolic and Neural Sciences, University of Modena and Reggio Emilia, Via del Pozzo 71, 41125 Modena, Italy; 3 Department for Life Quality Studies, Alma Mater Studiorum—University of Bologna, Corso d’Augusto, 47921 Rimini, Italy; cristina.angeloni@unibo.it

The great increase in life expectancy is linked to the necessity of counteracting chronic-degenerative diseases, e.g., cancer, metabolic syndrome, type 2 diabetes, cardiovascular, and neurodegenerative diseases that affect a high percentage of the population. 

Oxidative stress and inflammation are common features of these diseases and have different impacts on the function and structure of organs and tissues. The aim of this Special Issue was to collect original articles and reviews on this multifaceted and still largely unexplored topic in order to provide support for the development of novel potential therapeutic approaches.

**Bandari et al.** investigated the effects of chitin derivative compounds in alleviating lung inflammatory diseases such as acute lung injury (ALI), acute respiratory distress syndrome (ARDS) [1], and bronchopulmonary dysplasia (BPD) [2]. Although many efforts have been made to develop drugs to prevent or counteract these lung diseases, to date there are no effective cures available.

ALI/ARDS is a severe inflammatory pulmonary disease characterized by acute inflammation, microvascular damage, and increased pulmonary vascular and epithelial permeability, frequently resulting in acute respiratory failure and death. Bandari and coworkers, in a previous study [3], showed the anti-inflammatory effects of a novel small molecule AVR-25 derived from the chitin molecule (a long-chain polymer of N-acetylglucosamine). On these bases, the paper published in this Special Issue investigated the efficacy of two chitin-derived compounds, AVR-25 and AVR-48, in the LPS and hyperoxia-induced experimental ARDS in mice [1]. The treatment of mice with both AVR-25 and AVR-48 resulted in reduced lung inflammation, cell death, and improved pulmonary endothelial barrier function with overall recovery in pulmonary edema and injury. They also investigated the potential protective effect of AVR-48 in the CLP-induced mouse model of sepsis, as sepsis is another cause of ALI and ARDS. The compound triggered a reduction in mortality and overall injury of all organs (including the lung) in the CLP model. These data suggest that AVR-25 or AVR-48 could be potential therapeutic drugs for treating ALI/ARDS.

Bronchopulmonary dysplasia is a neonatal condition that occurs in infants born prematurely and with low birth weight. This disease is associated with an abnormal pulmonary function that may lead to BPD-associated pulmonary hypertension (PH), a major contributor to neonatal mortality and morbidity [4]. BPD is an inflammatory disease characterized by high levels of cytokines, chemokines, and inflammatory cells in the lungs and blood serum, and plasma. **Das et al.** [2] evaluated the safety and efficacy of AVR-48 as prophylactic therapy for preventing experimental BPD in a hyperoxia-induced experimental neonatal mouse model, using different routes of administration. AVR-48 was administered 2 and 4 days after birth. Pharmacokinetic and toxicity studies were carried out to ascertain bioavailability and safety. AVR-48 treatment led to an improvement of alveolar simplification, a reduction of inflammatory cells in bronchoalveolar lavage fluid, and a decrease in Fulton’s Index, lung inflammation, and cell death. Moreover, AVR-48 treatment increased angiogenesis and cell proliferation. Thanks to its safety and its ability to reverse the worsening cardiopulmonary phenotype of experimental BPD and BPD-PH, the authors suggest AVR-48 as a future drug candidate for the treatment of this condition.

Skeletal muscle damage is a common clinical manifestation of systemic sclerosis (SSc). In recent years, the role of local tissue cells (i.e., fibroblasts, cardiomyocytes, and myocytes) in the pathogenesis of the inflammatory events sustaining allo- or autoimmune-driven tissue inflammation and damage has been increasingly recognized [5,6]. In particular, human skeletal muscle cells under inflammatory stress have been shown to behave as an active counterpart, being a cellular source of CXCL10 (C-X-C chemokine ligand 10). In their study of diabetic cardiomyopathy, a co-morbid inflammation-driven condition, Corinaldesi, and coworkers have previously reported that CXCL10 is a pharmacologic target of sildenafil, a phosphodiesterase type 5 inhibitor (PDE5i). Therefore, the aim of the study in this Special Issue by **Corinaldesi et al.** [7] was to explore whether CXCL10 could be a target of sildenafil at the cellular level and to support the hypothesis of a potential increased scope of treatment with sildenafil in SSc. They analyzed the levels of CXCL10 in the sera of 116 SSc vs. 35 healthy subjects: CXCL10 sera levels were three times higher in SSc compared to healthy controls. Interestingly, sildenafil treatment was associated with lower CXCL10 sera levels. To investigate whether sildenafil could target the intracellular paths underlying CXCL10 release in human skeletal muscle cells under inflammatory stress they evaluated STAT1, JNK, and NF-kB phosphorylation in vitro. Indeed, a well-recognized function of NF-κB is the regulation of inflammatory responses and it is therefore unsurprising that deregulated NF-κB activation is a hallmark of chronic inflammatory disease. CXCL10 release was induced by IFNγ+TNFα in striated cells, while sildenafil dose-dependently reduced CXCL10 release by activated myocytes and impaired STAT1, NFκB, and JNK phosphorylation. In conclusion, there was a strong correlation between the level of serum CXCL10 and the clinical severity of muscle involvement. There was also a rise in creatine kinase (CK) serum concentration, suggesting a potential involvement in muscle damage in SSc. Indeed, sildenafil-induced CXCL10 inhibition at the systemic and human muscle cell level supports the hypothesis that PDE5i could be a potential therapeutic therapy to prevent and treat muscle damage in SSc.

The ROS/RNS (reactive oxygen and nitrogen species) pool from various sources may form a deleterious feedback circuit for melanomagenesis. Nevertheless, endogenous ROS can act as preventive agents for melanomagenesis as they kill damaged cells, but they can trigger cell proliferation and promote transformation. Thus, the function of ROS in melanomagenesis is indeed complicated, depending on the concentration and localization of ROS production [8]. Intracellular ROS can be generated by the NADPH oxidase family. It has been reported that Nox4 was up-regulated in more than half of melanoma cell lines tested and that a higher than normal expression of Nox4 was detected in at least one-third of melanoma patients’ samples, suggesting a correlation between Nox4 expression and melanoma development [8]. The findings hint that Nox4-generated ROS are required for the transformation phenotype of melanoma cells [9]. Moreover, several signaling pathways, including receptor tyrosine kinases, have a role in the development and progression of melanocytic lesions and malignant melanoma. Among these, the hepatocyte growth factor (HGF)/c-met axis emerges as a critical player because it can play a role in drug resistance. Indeed, 50% of melanoma patients present BRAF mutations, however, responders develop resistance to the inhibitors typically within one year of treatment. Interestingly, BRAF inhibitors induce ROS in melanoma cells. **Maraldi et al.** [10] examined how Nox4 derived ROS in BRAF mutated melanoma cells regulate their metastatic progression and drug sensitivity. At first, they analyzed Nox4 and c-met expression in early, late, and non-metastatic melanoma patients, then they investigated the cellular ROS modulation by HGF/c-met and Nox4 inhibition in vitro. One interesting outcome was the correlation between the high positivity for Nox4, c-met, and metastasis occurring at least 1 year later than melanoma diagnosis in patients with the BRAF mutation, in contrast to patients without the BRAF mutation. In vitro experiments demonstrated that the axis HGF/c-met/Nox4/ROS triggers the epithelial-mesenchymal transition, therefore, the data reported in this paper suggests a link between the HGF/c-met/Nox4 axis and metastatic progression through the epithelial-mesenchymal transition (EMT). Thus, the association of Nox4 inhibitors with the current therapy used to treat melanoma patients with BRAF mutations could be a good strategy to counteract the onset of metastasis.

In addition to these four research papers, this Special Issue offers comprehensive reviews on different aspects of the link between oxidative stress and chronic-degenerative disease and how to counteract them.

Starting from an animal model of diabetes mellitus, a very well-known disease linked to oxidative stress, **Salazar-García et al.** [11] analyze the advantages and disadvantages of currently available therapy on rodent animal models of DM, highlighting the potential anti-oxidative effects of natural compounds and their mechanisms of action. Animal models are indeed useful for exploring the cellular and molecular mechanisms of DM and improving novel therapeutics for their safe use in human beings. Regardless of the great potential of natural compounds in the management or treatment of DM in rodent models, there is limited information concerning their efficacy in human clinical trials. The antidiabetic activity of ginseng has been demonstrated in clinical trials [12]. Grape polyphenols prevented oxidative stress and insulin resistance in first-degree relatives of DM2 patients [13]. A moderate antidiabetic effect of cinnamon was observed in a randomized placebo-controlled clinical trial. *Allium cepa* L., also known as the bulb onion or common onion, has been shown to exhibit antidiabetic activity. Aloe vera extracts have been shown to exhibit a substantial decrease in glucose [14]. Finally, the authors show that some natural compounds could improve DM in clinical trials. However, more clinical trials and prospective, well-designed research are still needed to confirm these results.

**Yi-Hsuan et al.**, instead, showed how antioxidants can be useful in counteracting bladder hyperactivity induced by oxidative stress and bladder ischemia [15]. The occurrence of an overactive bladder, characterized by urinary frequency and urgency, nocturia, and urgency incontinence, has a significant negative impact on the quality of life of people with the condition and can cause withdrawal from social activities. A treatment that can at least reduce this symptomatology is therefore desirable. Since changes in the biomarkers of oxidative stress in overactive bladder animal models support the association between oxidative stress and urinary dysfunction and oxidative stress is related to increased bladder nerve activity and intravesical pressure [16], numerous studies implying the use of antioxidant and anti-inflammatory agents were conducted in animal model and reported in this review. In particular, epigallocatechin-3-gallate (EGCG), coenzyme Q10 (CoQ10), and melatonin were linked to a decrease in inflammation markers and an increase in antioxidant activity. However, in accordance with available studies, monotherapy with a single antioxidant agent may not be sufficient to reduce symptoms and obtain objective data in patients with overactive bladder. Therefore, a combination of therapies using antioxidants and other agents may be useful for those patients. Moreover, the Authors discuss the relationships between urinary dysfunction and oxidative stress biomarkers in urine, blood, and bladder tissue, suggesting the necessity of further studies in order to find treatment strategies for urinary dysfunction related to an overactive bladder.

The aim of the review written by **Bernatoniene et al.** [17] is to show and discuss the pleiotropic effects of isoflavones in Inflammation and chronic degenerative diseases. Isoflavones, such as genistein, daidzein, and glycitein, are secondary metabolites produced predominantly in leguminous plants endowed with phytoestrogenic and antioxidant properties due to high similarity to 17-β-estradiol together with a polyphenolic structure. The authors highlight the ability of Isoflavones to decrease inflammation, suppress oncogenic processes, and exert beneficial effects during aging and estrogen depletion. In particular, they reported the potential of isoflavones as bioactive compounds in alternative therapies for the treatment and prevention of hormone-related cancers. Moreover, isoflavones might be beneficial in reducing the risk of and/or alleviating metabolic diseases. Promising results also suggest that isoflavones could act as cardioprotective compounds, although more detailed studies are required to confirm their beneficial effects in humans. As far as neurodegenerative diseases are concerned, despite the neuroprotective effects of isoflavones observed in vitro in various cell cultures and in animal models, the results from the human clinical trials were contradictory [18] and more studies are needed to draw any conclusions about the preventive and beneficial effects of isoflavones against neurodegenerative diseases. Furthermore, in vitro and animal studies of the effects of isoflavones in rheumatoid arthritis suggest that these compounds could be used as a natural complementary treatment for this disease. Isoflavones have been shown to reduce menopausal symptoms like hot flashes, and this effect is linked to the estrogenic activity of isoflavones. Thus, they might be used for alleviating menopause-related symptoms without the risk of side effects related to conventional therapy.

Last but not least, in the review by **Silvestro et al.** [19] the antioxidant properties of curcuminoids in neurodegenerative diseases are analyzed and discussed in depth. Several in vitro and in vivo studies provide evidence of the anti-inflammatory and antioxidant properties [20] of curcuminoids, in particular of curcumin, Demethoxycurcumin, and Bisdemethoxycurcumin, obtained from the rhizomes of Curcuma Longa. It is well known that reactive oxygen species play a key role in the onset of neurodegenerative diseases such as Alzheimer’s disease, Parkinson’s disease, and amyotrophic lateral sclerosis (ALS). Therefore, numerous studies were performed in order to assess the potential role of curcuminoids in the prevention and counteraction of these typical chronic degenerative diseases. Curcuminoids are able to inhibit not only enzymes such as MAPK and c-JNK but also transcription factors (such as NF-κB, AP-1, Notch-1, β -catenin, and PPAR-γ) together with several pro-inflammatory cytokines. Moreover, this review highlights that curcuminoids exert powerful antioxidant actions through the activation of the Akt/Nrf2 pathway. Although several preclinical studies have shown that curcuminoids possess therapeutic efficacy against AD, PD, and ALS, clinical studies are needed in order to include curcuminoids in clinical practice.

Although not every chronic degenerative disease has been examined here, we earnestly hope that these findings will provide new ideas for strategies to counteract these diseases in the future. The second edition of this Special Issue, which is currently open to new submissions, will provide additional scientific evidence in order to contribute toward new understandings of chronic degenerative diseases and new preventive or o-treatment strategies.
